# Immersion, Absorption, and Spiritual Experience: Some Preliminary Findings

**DOI:** 10.3389/fpsyg.2020.02118

**Published:** 2020-09-02

**Authors:** Joseph Glicksohn, Tal Dotan Ben-Soussan

**Affiliations:** ^1^Department of Criminology, Bar-Ilan University, Ramat Gan, Israel; ^2^The Leslie and Susan Gonda (Goldschmied) Multidisciplinary Brain Research Center, Bar-Ilan University, Ramat Gan, Israel; ^3^Research Institute for Neuroscience, Education and Didactics, Patrizio Paoletti Foundation for Development and Communication, Assisi, Italy

**Keywords:** spiritual experience, mystical experience, EEG, alpha, absorption

## Abstract

Many traditions have utilized silent environments to induce altered states of consciousness and spiritual experiences. Neurocognitive explorations of spiritual experience can aid in understanding the underlying mechanism, but these are surprisingly rare. We present the verbal report and the electroencephalographic (EEG) alpha profile of a female participant scoring a maximal 34 on the Absorption Scale, recorded before and while she was immersed in a whole-body perceptual deprivation (WBPD) tank. We analyze her trancelike experience in terms of the imagery reported: a spaceship, corridors, doors, a man dressed in white, speaking to God, the sun, supernova, concentric images, and an out-of-body experience. Her report is indicative of a spiritual experience, given that she felt that she was “meeting God” in the laboratory. She exhibited both frontal and parietal left > right alpha power asymmetry at baseline, whereas in the WBPD condition, there was a global increase in alpha power and especially a sharp increase in right-frontal alpha power. Her verbal report and EEG alpha profile were compared to those of another female participant, also scoring high on absorption, whose verbal report was also indicative of a trancelike experience. For further comparison, we present the data for two participants scoring low on absorption. Spiritual experience that can be verbalized might be associated with a marked increase in right-frontal alpha power, as reported here. In contrast, a mystical experience characterized by ineffability would be indicated by a marked increase in left-frontal alpha power.

## Introduction

Many traditions have utilized silent environments to induce altered states of consciousness and spiritual experiences (Ustinova, 2017). Trait absorption, namely, the individual’s ability to fully engage attention in an experience, is a primary predictor of such spiritual or religious experience (Hunt, 2000; Luhrmann et al., 2010; Luhrmann, 2017; Lifshitz et al., 2019), in the same manner that this trait predisposes for affiliated experiences, such as hallucinations (Glicksohn, 2004), sensed presence (Granqvist et al., 2005), and altered states of consciousness (Glicksohn and Avnon, 1997–1998; Glicksohn, 2019). This is especially so when coupled with generated states of heightened absorption in an appropriate setting (Bronkhorst, 2016). As Glicksohn and Berkovich Ohana (2011, p. 54) have suggested, “the higher the absorption score, the more entranced the individual will be, circumstances permitting.” Absorption is typically assessed using Tellegen’s Absorption Scale (TAS; Tellegen, 1981).

A recent study employing the TAS and subjective experience of meditators, such as those of the present sample, has reported for them a mean score of 23, which was significantly higher than a mean score of 18 for non-meditating controls (Berkovich-Ohana and Glicksohn, 2017). Consequently, in the current study, we expected our participants to score relatively higher than a normative median of 19 (Glicksohn and Barrett, 2003). Further, as reported in that study, participants scoring 29+ comprised 5.6% of our normative sample; hence, the verbal reports of those participants in the present study who score between 29 and 34 are of primary interest for our investigation of spiritual experience.

In recent research looking at subjective experience arising from immersion in an environment comprising whole-body perceptual deprivation (WBPD; Glicksohn et al., 2017, 2019; Ben-Soussan et al., 2018), participants reported changes in both spatial and especially temporal experience, including reports of timelessness, which would be considered by some as constituting one component of religious experience (Fingelkurts, 2009). A stronger case could be made if some of the participants indicated that they had heard the voice of God or that they felt the presence of God (Fingelkurts, 2009). While these should be rare occurrences, they might certainly occur in participants with high trait absorption (Lifshitz et al., 2019), who experience high state absorption (or immersion) in their session (Glicksohn and Berkovich Ohana, 2012). The degree of endorsement of an item indicating having heard the voice of God ranges between 2 and 4% (Glicksohn and Barrett, 2003, p. 841). Reviewing those data involving a normative sample of 656 participants, we can now report that of 252 participants in that study who also completed the TAS, there were 12 who indicated such a spiritual experience, and of these, three scored between 29 and 34 on the TAS. In the present article, we shall report in detail the subjective experience of one of our participants, P2, presented in brief elsewhere (Glicksohn et al., 2019), who reported “meeting God” in the laboratory.

Following Lifshitz et al. (2019, p. 6), we classify her subjective report as being indicative of a *spiritual* experience. This is because in her verbal report, P2 fulfills the following three criteria: (1) “hearing God speak to you in a way you felt you heard outside your head”; (2) “having a vision that you felt was given to you by God”; (3) “feeling God near-tangibly present, as if he were standing there by your side.” Her report is not viewed as being indicative of a *mystical* experience, primarily because she does not explicitly say that her experience was “ineffable, incommunicable, and indescribable” (Stace, 1960, p. 55). There is, however, a commonality of experience; for following Stace (1960, p. 131), she fulfills the criterion of “feeling of the holy, sacred, or divine.”

Although neurocognitive explorations of spiritual experience can aid in the understanding of the underlying mechanism and have social implications, they are surprisingly rare (Fingelkurts, 2009; Le and Silverman, 2011). Presumably, to recall and perhaps to relive a mystical experience in the laboratory (Beauregard and Paquette, 2008) is not quite the same as to actually, and spontaneously, experience such an event. Describing such an altered state of consciousness can be revealing of the type of thought engaged in at the time (Glicksohn and Avnon, 1997–1998; Glicksohn and Lipperman-Kreda, 2007). The aim of the current article is to present and comment on such a rare spontaneous spiritual experience, as reported to us, and to investigate its electrophysiological correlates, focusing on the alpha band (8–12 Hz). In an altered state of consciousness, relative to a “resting wakefulness” state of consciousness, the left hemisphere is thought to become less active (hence, more alpha activity in that hemisphere), which is in turn balanced by a greater degree of activation of the right hemisphere. Thus, one would expect a shift to right-hemisphere dominance in altered states of consciousness (Davidson, 1976; Glicksohn and Berkovich Ohana, 2011). In addition, if the left hemisphere is less active, then the use of literal language and analytical thinking becomes impaired, leading in turn to both the experience of ineffability and a shift to an imagery-based mode of cognition (Glicksohn and Berkovich Ohana, 2011).

## Materials and Methods

### Participants and General Procedure

A total of 32 healthy participants participated in this study (labeled, as in Glicksohn et al., 2019, S1–S16, and P1–P16), all being experienced contemplative practitioners, chosen to participate because of their enhanced introspective and reporting abilities. The study was approved by the ethics committee of Bar-Ilan University. Upon entering the laboratory, the participant signed a written informed consent. They all completed a number of questionnaires prior to entering the WBPD chamber, including the TAS, of present interest. Prior to entering the chamber, the participants completed a time-production (TP) task (these data have been reported in Glicksohn et al., 2017). Then, a 5 min, eyes-closed electroencephalogram (EEG) baseline recording was obtained in the open WBPD chamber, prior to the closing of its door and its illumination with white light (5 min, eyes-closed condition, labeled in the figures as baseline). The white-illuminated WBPD chamber was then closed, and a 5 min, eyes-closed EEG recording (WBPD-1) allowed us to test for the immediate effect of the WBPD. This was followed by red and indigo light, each presented for 5 min (eyes-open conditions; these 2 colored-light conditions were presented in a counterbalanced order across participants), after which a short report of subjective experience was obtained (these data, both verbal protocols and EEG, have been reported in Glicksohn et al., 2019). Then, a third 5 min, eyes-closed EEG was recorded (WBPD-2), followed by a short report of subjective experience. At the end of the session, the participants completed a second TP task and underwent an extensive interview. In this article, we focus on four participants, P2, P15, S12, and P8, presenting both their subjective reports regarding their experience during WBPD and their EEG alpha profile.

### Whole-Body Perceptual Deprivation

**FIGURE 1:**
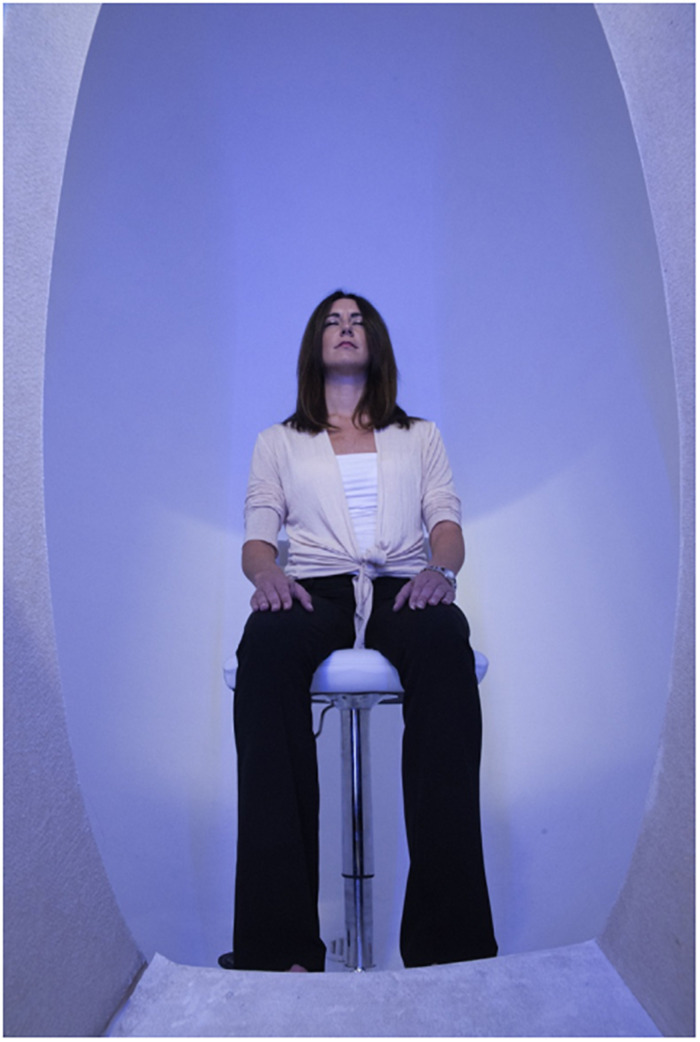
The OVO whole body perceptual deprivation (WBPD) tank located in the Research Institute for Neuroscience, Education and Didactics (RINED) of the Paoletti Foundation. Written informed consent was obtained from the individual for the publication of the potentially identifiable images or data included in this article.

The OVO WBPD chamber (in the shape of an egg; see Glicksohn et al., 2017, 2019; Ben-Soussan et al., 2018; Figure 1), created by Patrizio Paoletti and based on his Sphere Model of Consciousness (Paoletti, 2002; Paoletti and Ben-Soussan, 2019; Paoletti and Soussan, 2020), is located in the Cognitive Neurophysiology Laboratory, at the Research Institute for Neuroscience, Fondazione Patrizio Paoletti, Assisi, Italy. The chamber was flooded with white light, followed by red light and indigo light (in a counterbalanced order across participants), enabling a totally immersive WBPD. In such an environment, the participant sits in isolation and in silence. The participant’s verbal reports were heard through a microphone and were recorded.

### Assessment of Trait Absorption

The TAS (Tellegen, 1981) comprises 34 true/false items that participants complete regarding their cognitive–affective subjective experience (e.g., with respect to synesthesia).

### EEG Recording and Analyses

EEG was recorded using a 65-channel geodesic sensor net (Electrical Geodesics Inc., Eugene, OR, USA) at a 500 Hz sampling rate, referenced to the vertex (Cz), with analog 0.1–200 Hz bandpass filtering. Impedance was kept under 40 kΩ, which is within the accepted range for this system. The data were subsequently referenced offline to average reference, and both preprocessing of the data and subsequent spectral analysis (within the range of frequencies from 1 to 45 Hz) were conducted – all in line with our previous publications (e.g., Berkovich-Ohana et al., 2012; Ben-Soussan et al., 2014). In the full report of these data (Glicksohn et al., 2019), we focused on eight regions of interest (ROIs), enabling us to present an EEG alpha profile for each hemisphere, spanning the longitude extending from frontal (F), to central (C), to parietal (P), and to occipital (O) sites. On average, there were ∼40 two-second epochs of eyes-closed EEG data for each section of time – beginning, middle, end – of each condition (baseline, WBPD-1, WBPD-2), each of which was subjected to spectral analysis. Power values were summed across bins, to derive alpha (8–12 Hz) power, which was subsequently log-transformed to normalize the data. Analysis of the EEG alpha profile was conducted using a repeated-measures analysis of variance: condition (baseline, WBPD-1, WBPD-2) × time (beginning, middle, end) × hemisphere (left, right) × ROI (F, C, P, O). The analysis is conducted at the *individual* level (*n* = 1); hence, one needs to define a suitable error term. To do this, the sum of squares (SS) for all higher-order interaction terms was pooled, which we subsequently labeled as *SSE*, as was their *df* (*dfE* being 40). Focusing on the six two-way interactions, we adopted a Bonferroni-corrected *p*-value of 0.008 (0.05/6) for each of these. Table 1 (derived from a larger table appearing in Glicksohn et al., 2019) summarizes the main results for each of the four participants reported here (P2, P15, S12, P8).

**TABLE 1:** Individual EEG alpha profiles, specifying three two-way interactions that are significant for one or more of the participants.

			**Hem × ROI**		**Condition × Hem**		**Condition × ROI**	
				
**Individual**	**Gender**	**Age (y)**	***F*(3, 40)^a^**	**Partial η^2^**	***F*(2, 40)^b^**	**Partial η^2^**	***F*(6, 40)^c^**	**Partial η^2^**
P2	F	30	40.29*	0.75	10.43*	0.34	3.77*	0.35
P15	F	44	46.15*	0.78	4.00		3.38	
S12	M	46	5.11*	0.28	4.53		2.66	
P8	M	44	14.62*	0.52	1.66		1.93	

Hem, hemisphere; ROI, region of interest. Critical values for *corrected p-value (0.008): ^*a*^F_*crit*_ = 4.98; ^*b*^F_*crit*_ = 6.07; ^c^F_*crit*_ = 3.71.

### Analysis of Individual EEG Alpha Profiles

In the present report, we focus on the frontal and parietal components of this EEG alpha profile. We thus examine 12 power values for each individual: left frontal (LF), right frontal (RF), left parietal (LP), and right parietal (RP) at baseline, at WBPD-1, and at WBPD-2. For each site (LF, RF, LP, RP) we define two orthogonal contrasts: (1) comparing WBPD-2 to WBPD-1, allowing us to assess the influence of WBPD on alpha power, and (2) comparing the average of WBPD-2 and WBPD-1 to baseline, allowing us to assess the difference between baseline and WBPD. For these contrasts, we use *SSE* from the complete profile as a suitable error term. In addition to these contrasts, an inspection of alpha asymmetry (e.g., L > R, or F > P) should be correlated with particular reports of subjective experience (see below), as follows: (1) positive (LF < RF) or negative (LF > RF) affect (e.g., Davidson, 1992); (2) trancelike (F > P) or reflective (F < P) state of consciousness (Glicksohn and Berkovich Ohana, 2011).

### Analysis of Subjective Reports

The subjective reports, which included both open-ended descriptions of the experience during the session and answers to the semistructured interview conducted at the end of the session, were given to five independent judges, having no familiarity with the participants. In the full report of these data (Glicksohn et al., 2019), we focused on three categories: *valence* (positive vs. negative), *mode of thinking* (verbal vs. imagistic), and s*tate of consciousness* (reflective vs. trancelike). The judges were given the printed reports, together with a coding sheet specifying these categories, and had to make decisions for each participant and for each category. In particular, a reflective state of consciousness is defined by an act of reflective awareness on ongoing experience, whereas a trancelike state of experience is defined by the lack of reflective awareness (e.g., Glicksohn and Berkovich Ohana, 2011; Glicksohn and Berkovich Ohana, 2012). Participants P2 and P15 were considered to have reported a trancelike experience by at least four of the five judges. P2, in particular, reported a spiritual experience.

## Results

### Verbal Reports

Our focus is on the subjective experience of participant P2, who spontaneously reported “meeting God” in the laboratory. We first present her report, and then for comparison those of three other participants: P15, S12, and P8. We comment on the reports of P2 and P15, trying to contextualize the imagery of their trancelike experiences.

#### Case 1

Participant P2 is a healthy, right-handed 30-year-old woman, with no history of psychological or physical trauma or substance abuse, scoring 34 (out of 34) on the TAS. Her subjective report (appearing abridged in Glicksohn et al., 2019) is the single report we have that, to our mind, could be clearly indicative of a spiritual experience. Here is her report, which was found to depict a trancelike experience by our independent judges (Glicksohn et al., 2019):

…there was this spaceship that was carrying me around the universe. When the blue light set in, I saw several corridors that lead me to several doors, which could be open, and at the end there was a person dressed in white who welcomed me, and I said to myself: “I’m meeting God.” [*laughing*]

(Exp: “Have you ever had a similar experience?”)

To meet him in person, no. But, to speak to him, yes. It was like going back home, and he said to me, “So you remember why you are on Earth now? Do you know what you have to do? You have just to continue to shine and bring light to people.” That’s what I heard when he appeared before me.

Afterward, I heard the sounds, and it was like going back into the spaceship and going around, and I felt I have to move on [*laughing*]. When the red light set in, I felt like I was in the sun and that there was this sun that illuminated everything, and I thought I have to do the same, without making distinctions, but just to shine and to be calm [*sighs*].

There was this light that was like a supernova, when planets collide, and they produce many colors. I could see many concentric images, and to travel in the universe, in this vast space. Afterward, the white light appeared again, and I came back here into the egg.

(Exp: “Have you ever had a similar experience?”)

I had similar experiences during meditation, with me leaving the body and going around into space. It was like a journey between the microcosm of my body and the macrocosm of the universe. I thought I was able to let the sun come in once I was out of the egg [*laughing*].

A few words regarding the content of this participant’s report are in order, especially in relation to (1) dreams, (2) passages and overcoming obstacles, (3) encounters with higher beings, as we will detail in the following paragraphs.

(1) Dreams – The mention of a *spaceship* brings to mind Jung (1978, p. 63), who writes when describing a dream of “… a space-ship that comes out of the beyond to the edge of our world in order to fetch the souls of the dead. It is not clear from the vision where the ship comes from….”
(2) Passages and overcoming obstacles—The participant continues with a report of seeing “several corridors that lead to several doors,” which is in line with Hume’s (2007, p. 6) suggestion that “The notion of getting through an obstacle, or having a passage open up to permit entry to another, more sacred dimension, permeates myths, legends, religious writing and personal narrations throughout history… but the passage from one to the other requires opening some sort of portal. Expressions such as ‘gate,’ ‘way,’ ‘door,’ ‘ladder,’ ‘bridge,’ have been employed in religious discourse and texts to indicate that movement is indeed possible.”
(3) Encounters with higher beings – The figure in white, which she reports encountering, appears in various such accounts. For example, Shanon (2002, p. 107) writes: “At one turn of the trail I met a bearded old man sitting on a beautifully adorned chair. The man wore a splendid white robe full of rich embroidery, some of it golden, held a scepter in his hand and his countenance was wise and benevolent.” This encounter is further in line with a rich body of literature related to (3.1) near-death experiences (NDEs), (3.2) mystical descriptions of God, and (3.3) the unity between the microcosmos and the macrocosmos, as will be detailed below.

(3.1) In an NDE in which there are reports of both timelessness (Wittmann et al., 2017) and “meeting God” (as seen in the web-based databank of the Near Death Experience Research Foundation, http://www.nderf.org/index.htm, employed by Wittmann et al., 2017), a characteristic account of such an experience would be “I encountered a form, who I knew was God, who told me it was time to now go back”^1^. In the verbal report of P2, God has a mission for her. Following Lifshitz et al. (2019, p. 6), we classify her subjective report as being indicative of a *spiritual* experience, because she explicitly reported “meeting God” in the laboratory.
(3.2) The notion of shining like a sun, both literally and metaphorically, is familiar from the mystical literature. For example, Underhill (1955, p. 237) writes: “…and straightway he became all shining like the sun.” Stace (1960, p. 97) notes “That God is light is the common metaphor for his goodness and blessedness.” Further, Underhill (1955, p. 249) writes, “A new sun rises above the horizon, and transfigures their twilit world. Over and over again, they return to light-imagery in this connection.”
Her imagery of concentric images could have certainly been influenced by various science fiction movies that have appeared over the years and especially by Stanley Kubrick’s adaptation of Arthur. C. Clarke’s, *2001: A Space Odyssey*^2^. There are, however, other precursors. Watts (1962, p. 29), for example, provides an earlier and comparable description: “I begin to feel that the world is at once inside my head and outside it, and the two, inside and outside, begin to include or ‘cap’ one another like an infinite series of concentric spheres.” The concentric circles are, perhaps “images of phosphene rings (annuli), and images of amorphous expanding waves” (Nicholson and Firnhaber, 2004, p. 56).
Similarly to NDEs, out-of-body experiences are occasionally reported by experienced meditators (Berkovich-Ohana et al., 2013). Suedfeld (1980, p. 204) has made the insightful comment: “One problem with the scientific paradigm followed by most workers in this area is that it tends to concentrate on group results and quantitative data, so that such occurrences usually do not appear in the published research even when they have been mentioned by subjects.” In the present article, we are attempting to rectify this.
(3.3) Finally, the journey the participant describes between the microcosm of her body and the macrocosm of the universe, reminds one of Jung’s (1978, p. 29) writing related to: “… psychic wholeness, as the historical testimonies show, has always been characterized by certain cosmic affinities: the individual soul was thought to be of “heavenly” origin, a particle of the world soul, and hence a microcosm, a reflection of the macrocosm.”

#### Case 2

For comparison, participant P15 is a 44-year-old woman, scoring 32 on the TAS. Her subjective report is also colorful, but does not necessarily depict a spiritual experience, although this was also judged to present a trancelike experience by our independent judges (Glicksohn et al., 2019):

The first mental link I had was about being in my mother’s stomach, this relationship came to me, so that everything I experienced was what I would feel in the mother’s womb, and I heard the sounds as they were amplified, and these were the sounds produced by me; these were my sounds. So, I had a higher sensitivity for those of my breath, of the sound of my breath, all my hearing abilities were activated.

It was a very strong sensation, very intense from the sensory point of view. When the red set in, it had a very strong impact on me [*laughing*], and therefore there could have been a moment of anxiety in the red, but then I linked it to the sun on the stomach, to the belly exposed to light, and so the sun enters into the mother’s womb, this light….

In the blue, I really found my depth, I really felt myself, my deep being. It was linked to the earthly experience of the sea of the blue. However, apart from this mental link, the visual association of an ocean… I really felt my deep being, and this gave me a feeling of calm and peace and a sense of well-being, of self-collection [*sighs*].

When the white set in, I had temporal perception, because in the white I had the clear sensation of being inside the egg, of the spatial form, the sensory perception of the egg. But, at the same time, there was also a feeling of expansion. Concerning time, time was neither fast nor slow, but I stayed firm. There was absence of time… but, together with this, as I was moving in time, that is cued by my breath, time was cued by my sensations, but in reality, this is in the present, it wasn’t fast or slow, it just was what it was. To describe this… floating, in suspension, that is how I would describe it.

P15 reports womb-like imagery and other associations, which are encouraged by immersion in our WBPD, as in other comparable set-ups. For example, Benson (2001, p. 125) reports that observers placed within a Turrell Ganzfeld sphere frequently described their experience as being “calming, relaxing, womb-like, uplifting, meditative, and so on.” Experienced meditators, such as P15, also report “…like being in the mother’s womb. A deep state of ease and peace” (Travis et al., 2017, p. 162). She also refers to the sun, but in addition she addresses the colors. The notion that red light might influence both mood and subjective experience has a venerable history (Elliot et al., 2007).

#### Case 3

While no participant reported what would be considered to be a mystical experience, as defined by consensual criteria (Stace, 1960), a somewhat minor variant of the experience of unity was reported by participant S12, who is a 46-year-old man, scoring 24 on the TAS:

Very interesting, the sensation that everything is mind. There are no points of reference. Everything is the egg. It’s the environment, which is unfamiliar, and then I listen more to myself. Paid more attention to myself and to the noises of the body and of the stomach. They were expanded. It was weird and funny. It is an opportunity to look inside.

#### Case 4

For comparison, another 44-year-old man, P8, scoring 19 on the TAS, reported:

…I was very focused on my inner experience. I was very relaxed, to the point that at some stage I was actually on the edge between being awake and being asleep…. I saw images coming and going… I always had the feeling to be with a divided attention, one focused on the inside and one focused on what was going on outside….

### EEG Alpha Profiles

#### Case 1

**FIGURE 2:**
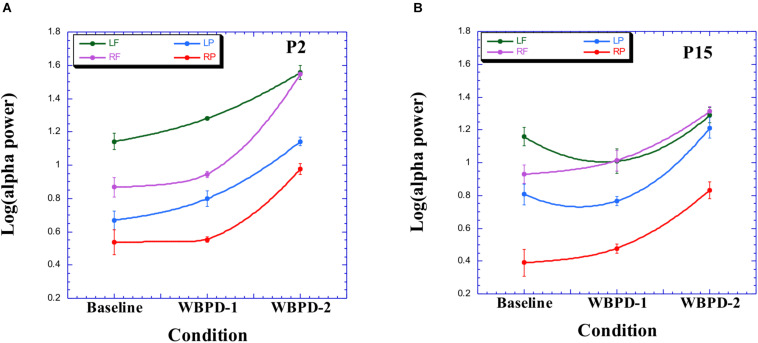
Alpha profile for participant P2 **(A)** and for P15 **(B)**. LF, left frontal; RF, right frontal; LP, left parietal; RP, right parietal. On average, there were ∼40 two-second epochs for each section of time – beginning, middle, end – of each condition (baseline, WBPD-1, WBPD-2), each epoch being subjected to spectral analysis. Derived power values within the alpha (8–12 Hz) band were then log-transformed and averaged, for each section of time within each condition. Mean values reported here are collapsed over time within condition. Error bars refer to SE within condition, based on the three computed means.

Participant P2 exhibited both frontal and parietal L > R alpha asymmetry (Figure 2A). In addition, note that she exhibits a sharp increase in right-frontal (RF) alpha power in the WBPD condition. In fact, as one reviewer has stressed, there is for her a global increase in alpha. Table 2 summarizes the individual orthogonal contrasts employed to help interpret her data (and those of the other three participants reported here), and as can be readily seen there, the size of effect for RF is impressive. Given that she reports having experienced a spiritual experience during WBPD, it is possible that this experience is associated with this marked increase in RF alpha power. As the same reviewer has suggested, at WBPD-2, the increase in RF alpha power now equals that of LF. When the chamber was flooded with white light the second time, P2 reports that she “came back here into the egg.” In fact, note that at this point in time, the L > R frontal alpha asymmetry is abolished. The parietal L > R alpha asymmetry has a corresponding focus on external space in the verbal report (spaceship, doors, etc.). P2 is completely immersed in this trancelike experience.

**TABLE 2:** Individual orthogonal contrasts.

**Individual**	**MSE**	**ROI**	**SS(WBPD-2 vs. WBPD-1)**	***F*(1, 40)**	**Partial η^2^**	**SS (WBPD vs. baseline)**	***F*(1, 40)**	**Partial η^2^**
P2	0.003	LF	0.038	10.96*	0.24	0.051	14.77*	0.30
	0.003	RF	0.182	52.87*	0.60	0.096	27.76*	0.44
	0.003	LP	0.059	17.15*	0.33	0.060	17.51*	0.33
	0.003	RP	0.091	26.30*	0.43	0.034	9.87*	0.22
P15	0.003	LF	0.039	11.44*	0.25	0.000	0.02	
	0.003	RF	0.045	12.95*	0.27	0.036	10.45*	0.23
	0.003	LP	0.099	28.82*	0.45	0.022	6.33	0.15
	0.003	RP	0.063	18.26*	0.34	0.047	13.52*	0.28
S12	0.052	LF	0.009	0.16		0.003	0.05	
	0.052	RF	0.225	4.30		0.073	1.39	
	0.052	LP	0.002	0.04		0.443	8.48	
	0.052	RP	0.001	0.02		0.209	3.99	
P8	0.004	LF	0.002	0.53		0.010	2.83	
	0.004	RF	0.018	4.98		0.013	3.66	
	0.004	LP	0.003	0.80		0.006	1.52	
	0.004	RP	0.001	0.22		0.000	0.00	

ROI, region of interest; LF, left frontal; RF, right frontal; LP, left parietal; RP, right parietal. *Given 8 contrasts for each individual, the p-value adopted for each = 0.006 (F_*crit*_ = 8.53).

#### Case 2

Participant P15 exhibits parietal (but not frontal) L > R alpha asymmetry during WBPD (Figure 2B). In fact, at WBPD-1, the L > R frontal alpha asymmetry is abolished. Thus, while P2 and P15 present similar parietal alpha profiles, frontally they diverge. For P15, while in the WBPD condition, there is no L > R frontal alpha asymmetry, primarily due to the gradual increase in RF alpha power, coupled with the decrease in LF alpha power in the transition from baseline to WBPD-1 (and note from Table 2 that there is no significant difference between baseline and WBPD for LF). As for P2, the parietal L > R alpha asymmetry has a corresponding focus on external space in the verbal report of P15 (“experience of the sea of blue”). P15 is completely immersed in this trancelike experience.

#### Case 3

**FIGURE 3:**
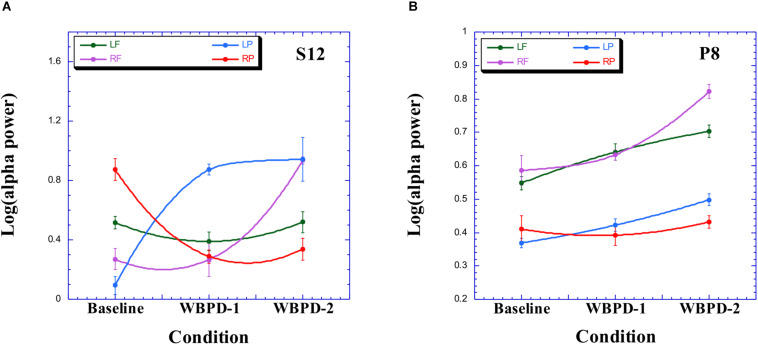
Alpha profile for participant S12 **(A)** and for P8 **(B)**. LF, left frontal; RF, right frontal; LP, left parietal; RP, right parietal.

Participant S12 has R > L parietal alpha asymmetry, which reverses (L > R) during WBPD (Figure 3A). There is a corresponding focus on external space in the verbal report (“Everything is the egg”). In addition, the L > R frontal alpha asymmetry also reverses during WBPD, primarily due to an increase of RF alpha power, which is somewhat similar to that observed for P2 (Figure 2A). Note from Table 2, however, that none of the contrasts are significant (the increase in alpha power between baseline and WBPD for LP does not pass our Bonferroni-corrected criterion).

#### Case 4

P8 also exhibits a reversal in hemispheric asymmetry, from parietal R > L at baseline, to L > R during WBPD (Figure 3B). There is a corresponding focus on external space in the verbal report (“one focused on what was going on outside”). Again, we note an increase of RF alpha power during WBPD, although as with S12, none of the contrasts are significant. Table 3 presents individual profiles for each of these participants, based on three sources of data: Trait absorption (TAS), subjective experience, and EEG alpha profile.

**TABLE 3:** Participant’s trait absorption (TAS), subjective experience, and EEG alpha profile.

			**Spiritual**	**Trancelike**	**Frontal**	**Parietal**	**During**
**Participant**	**Absorption**	**Ineffability**	**experience**	**experience**	**asymmetry**	**asymmetry**	**WBPD**
P2	High		+	+	L>R	L>R	RF
P15	High			+		L>R	LP
S12	Medium						
P8	Medium						

## Discussion

The present article adds to a small literature addressing neurocognitive explorations of spiritual experience (Fingelkurts, 2009; Le and Silverman, 2011). Trait absorption is clearly a relevant factor here, as others have also noted (e.g., Studerus et al., 2012). When Beauregard et al. (2009) requested from their participants “to mentally visualize and emotionally connect with the ‘being of light’ allegedly encountered” during their NDE, they recorded higher alpha power, relative to a control condition of mentally visualizing “the light emitted by a lamp,” in both hemispheres, frontally.

Here, we found that spiritual experience that can be verbalized might be associated with a marked increase in RF alpha power (P2), and this is worthy of further study. Thus, while there is a global increase in alpha for P2 reporting a spiritual experience, note from Table 2 the very large effect size for RF in particular.

We can contextualize these changes in alpha power and alpha asymmetry reported here for P2 and S12 with the aid of the literature, which discusses affect and alpha activity. First, note that the experience reported by P2 is both spiritual and positive. A positive experience should be correlated with lower LF alpha power, and a negative experience should be correlated with lower RF alpha activity (e.g., Davidson, 1992). If participants exposed to WBPD have sustained alertness and introspective sensitization, as one would expect from those individuals trained in contemplative meditation whose data we report here (such as P2), then one would also expect to see higher RP alpha (Benedek et al., 2014), implicating internally oriented attention. The state of consciousness of the participant can be either more trancelike (F > P) or more reflective (F < P), as we have argued (Glicksohn and Berkovich Ohana, 2011). Furthermore, Lodder and van Putten (2013, p. 230–231) have suggested categorizing the alpha power distribution as follows: (1) “normal,” if P > F; (2) “moderate differentiation,” if P = F; and (3) “deviant,” if F > P. Combining these notions, and looking at the individual alpha profiles, we note that both P2 and P15 display “deviant” differentiation at both baseline and during WBPD, and they were both immersed in their trancelike experience. But P2 reports a spiritual experience, which she has no problem in describing. Hence, the experience is not characterized by ineffability, as would be characteristic of a mystical experience. We stress that her spiritual (but not mystical) experience might be indexed by a marked increase in RF alpha power.

Mystical experience, which is characterized by ineffability (Stace, 1960), should be associated with a marked increase in LF alpha power (Davidson, 1976), and this should be looked for. A trancelike experience is associated with parietal L > R alpha asymmetry (Glicksohn and Berkovich Ohana, 2011), as reported in this article. A reversal in alpha asymmetry noted here, from parietal R > L at baseline, to L > R during WBPD (participants P8, S12), may well be indicative of the type of dynamic shift in hemispheric asymmetry predicted by Suedfeld et al. (1994) for exposure to our WBPD environment. Indeed, our use of WBPD facilitated what Travis et al. (2017, p. 162) have termed “lively silence,” which is “not passive or inert” – as clearly seen in the imagery reported by our participants. As McGilchrist (2019, p. 330) has recently noted, “A constant feature of all mystical traditions is an emphasis on the creative power of silence and stillness” and further that this facilitates “the mode of attention to the world [more] of the RH than the LH.” Our data – both experiential and electrophysiological – support this view.

## Data Availability Statement

The raw data supporting the conclusions of this article will be made available by the authors, without undue reservation, to any qualified researcher.

## Ethics Statement

Written informed consent was obtained from the individual for the publication of the potentially identifiable images or data included in this article.

## Author Contributions

JG and TB-S designed the research, analyzed the first-person reports and EEG data, and wrote the manuscript. Both authors contributed to the article and approved the submitted version.

## Conflict of Interest

The authors declare that the research was conducted in the absence of any commercial or financial relationships that could be construed as a potential conflict of interest.

## Footnotes

1 https://www.nderf.org/joann_m’s_nde.htm

2 https://en.wikipedia.org/wiki/2001:_A_Space_Odyssey_(novel)

## References

[B1] Beauregard M. Courtemanche J. Paquette V. (2009). Brain activity in near-death experiencers during a meditative state. *Resuscitation* 80 1006–1010. 10.1016/j.resuscitation.2009.05.006 19573975

[B2] Beauregard M. Paquette V. (2008). EEG activity in Carmelite nuns during a mystical experience. *Neurosci. Lett.* 444 1–4. 10.1016/j.neulet.2008.08.028 18721862

[B3] Benedek M. Schickel R. J. Jauk E. Fink A. Neubauer A. C. (2014). Alpha power increases in right parietal cortex reflects focused internal attention. *Neuropsychologia* 56 393–400. 10.1016/j.neuropsychologia.2014.02.010 24561034 PMC3989020

[B4] Benson C. (2001). “Points of view and the visual arts: James Turrell, Antonio Damasio and the “no point of view” phenomenon,” in *Theoretical Issues in Psychology*, eds Morss J. R. Stephenson N. van Rappard H. (New York, NY: Springer), 119–129. 10.1007/978-1-4757-6817-6_11

[B5] Ben-Soussan T. D. Berkovich-Ohana A. Glicksohn J. Goldstein A. (2014). A suspended act: increased reflectivity and gender-dependent electrophysiological change following Quadrato Motor Training. *Front. Psychol.* 5:55. 10.3389/fpsyg.2014.00055 24550872 PMC3909823

[B6] Ben-Soussan T. D. Mauro F. Lasaponara S. Glicksohn J. Marson F. Berkovich-Ohana A. (2018). Fully immersed: state absorption and electrophysiological effects of the OVO whole-body perceptual deprivation chamber. *Prog. Brain Res.* 244 165–184. 10.1016/bs.pbr.2018.10.023 30732836

[B7] Berkovich-Ohana A. Dor-Ziderman Y. Glicksohn J. Goldstein A. (2013). Alterations in the sense of time, space and body in the mindfulness-trained brain: a neurophenomenologically-guided MEG study. *Front. Psychol.* 4:912. 10.3389/fpsyg.2013.00912 24348455 PMC3847819

[B8] Berkovich-Ohana A. Glicksohn J. (2017). Meditation, absorption, transcendent experience and affect: tying it all together by the Consciousness State Space (CSS) model. *Mindfulness* 8 68–77. 10.1007/s12671-015-0481-9

[B9] Berkovich-Ohana A. Glicksohn J. Goldstein A. (2012). Mindfulness-induced changes in gamma band activity - implications for the default mode network, self-reference and attention. *Clin. Neurophysiol.* 123 700–710. 10.1016/j.clinph.2011.07.048 21940201

[B10] Bronkhorst J. (2016). Can religion be explained? The role of absorption in various religious phenomena. *Method Theory Study Relig.* 29 1–30. 10.1163/15700682-12341375

[B11] Davidson J. M. (1976). The physiology of meditation and mystical states of consciousness. *Perspect. Biol. Med.* 19 345–380. 10.1353/pbm.1976.0042 958856

[B12] Davidson R. J. (1992). Anterior cerebral asymmetry and the nature of emotion. *Brain Cogn.* 20 125–151. 10.1016/0278-2626(92)90065-T 1389117

[B13] Elliot A. J. Maier M. A. Moller A. C. Friedman R. Meinhardt J. (2007). Color and psychological functioning: the effect of red on performance attainment. *J. Exp. Psychol. Gen.* 136 154–168. 10.1037/0096-3445.136.1.154 17324089

[B14] Fingelkurts A. A. (2009). Is our brain hardwired to produce God, or is our brain hardwired to perceive God? A systematic review on the role of the brain in mediating religious experience. *Cogn. Proc.* 10 293–326. 10.1007/s10339-009-0261-3 19471985

[B15] Glicksohn J. (2004). Absorption, hallucinations, and the continuum hypothesis. *Behav. Brain Sci.* 27 793–794. 10.1017/S0140525X04280189

[B16] Glicksohn J. (2019). Patterns of occurrence of four states of consciousness as a function of trait Absorption. *J. Pers. Oriente. Res.* 5 27–36. 10.17505/jpor.2019.03 PMC7842639 33569139

[B17] Glicksohn J. Barrett T. R. (2003). Absorption and hallucinatory experience. *Appl. Cogn. Psychol.* 17 833–849. 10.1002/acp.913

[B18] Glicksohn J. Berkovich Ohana A. (2011). From trance to transcendence: a neurocognitive approach. *J. Mind Behav.* 32 49–62.

[B19] Glicksohn J. Berkovich Ohana A. (2012). “Absorption, immersion, and consciousness,” in *Video Game Play and Consciousness*, ed. Gackenbach J. (New York, NY: Nova Science Publishers, Inc), 83–99.

[B20] Glicksohn J. Berkovich-Ohana A. Mauro F. Ben-Soussan T. D. (2017). Time perception and the experience of time when immersed in an altered sensory environment. *Front. Hum. Neurosci.* 11:487. 10.3389/fnhum.2017.00487 29056902 PMC5635043

[B21] Glicksohn J. Berkovich-Ohana A. Mauro F. Ben-Soussan T. D. (2019). Individual EEG alpha profiles are gender-dependent and indicate subjective experiences in whole-body perceptual deprivation. *Neuropsychologia* 125 81–92. 10.1016/j.neuropsychologia.2019.01.018 30711610

[B22] Glicksohn J. Lipperman-Kreda S. (2007). Time, thought, and consciousness. *J. Mind Behav.* 28 289–305.

[B23] Glicksohn J. Avnon M. (1997–1998). Explorations in virtual reality: absorption, cognition and altered state of consciousness. *Imaginat. Cogni. Personal.* 17 141–151. 10.2190/FTUU-GLC5-GBT8-9RUW 22612255

[B24] Granqvist P. Fredrikson M. Unge P. Hagenfeldt A. Valind S. Larhammar D. (2005). Sensed presence and mystical experiences are predicted by suggestibility, not by the application of transcranial weak complex magnetic fields. *Neurosci. Lett.* 379 1–6. 10.1016/j.neulet.2004.10.057 15849873

[B25] Hume L. (2007). *Portals: Opening Doorways to Other Realities Through the Senses.* Oxford: Berg.

[B26] Hunt H. T. (2000). Experiences of radical personal transformation in mysticism, religious conversion, and psychosis: a review of the varieties, processes, and consequences of the numinous. *J. Mind Behav.* 21 353–397.

[B27] Jung C. G. (1978). *Flying Saucers: A Modern Myth of Things Seen in the Skies.* Princeton, NJ: Princeton University Press.

[B28] Le C. Silverman D. H. S. (2011). Neuroimaging and EEG-based explorations of cerebral substrates for suprapentasensory perception: a critical appraisal of recent experimental literature. *Psychiatry Res.* 194 105–110. 10.1016/j.pscychresns.2011.05.002 21924873

[B29] Lifshitz M. van Elk M. Luhrmann T. M. (2019). Absorption and spiritual experience: a review of evidence and potential mechanisms. *Conscious. Cogn.* 73:102760. 10.1016/j.concog.2019.05.008 31228696

[B30] Lodder S. S. van Putten M. J. A. M. (2013). Quantification of the adult EEG background pattern. *Clin. Neurophysiol.* 124 228–237. 10.1016/j.clinph.2012.07.007 22917580

[B31] Luhrmann T. M. (2017). Diversity within the psychotic continuum. *Schizophr. Bull.* 43 27–31. 10.1093/schbul/sbw137 27872266 PMC5216862

[B32] Luhrmann T. M. Nusbaum H. Thisted R. (2010). The absorption hypothesis: learning to hear God in evangelical christianity. *Am. Anthropol.* 112 66–78. 10.1111/j.1548-1433.2009.01197.x

[B33] McGilchrist I. (2019). Cerebral lateralization and religion: a phenomenological approach. *Relig. Brain Behav.* 9 319–339. 10.1080/2153599X.2019.1604411

[B34] Nicholson P. T. Firnhaber R. P. (2004). “Autohypnotic induction of sleep rhythms generates visions of light with form-constant patterns,” in *Shamanism in the Interdisciplinary Context*, eds Leete A. Firnhaber R. P. (Boca Raton, FL: BrownWalker Press), 56–83.

[B35] Paoletti P. Ben-Soussan T. D. (2019). The sphere model of consciousness: from geometrical to neuro-psycho-educational perspectives. *Log. Univ.* 13 395–415. 10.1007/s11787-019-00226-0

[B36] Paoletti P. (2002). Flussi, territori, luogo [Flows, territories, place]. M.E.D. Ed. Madeira: M.E.D. Publishing.

[B37] Paoletti P. Soussan T. D. B. (2020). Reflections on silence and consciousness without contents according to the Sphere Model of Consciousness. *Front. Psychol.* 10.3389/fpsyg.2020.01807 PMC7435012 32903475

[B38] Shanon B. (2002). *The Antipodes of the Mind: Charting the Phenomenology of the Ayahuasca Experience.* Oxford: Oxford University Press.

[B39] Stace W. T. (1960). *Mysticism and Philosophy.* London: Macmillan & Co Ltd.

[B40] Studerus E. Gamma A. Kometer M. Vollenweider F. X. (2012). Prediction of psilocybin response in healthy volunteers. *PLoS One* 7:e30800. 10.1371/journal.pone.0030800 22363492 PMC3281871

[B41] Suedfeld P. (1980). *Restricted Environmental Stimulation: Research and Clinical Applications.* New York, NY: John Wiley.

[B42] Suedfeld P. Steel G. D. Wallbaum A. B. C. Bluck S. Livesey N. Capozzi L. (1994). Explaining the effects of stimulus restriction: testing the dynamic hemispheric-asymmetry hypothesis. *J. Environ. Psychol.* 14 87–100. 10.1016/S0272-4944(05)80163-X

[B43] Tellegen A. (1981). Practicing the two disciplines for relaxation and enlightenment: comment on “Role of the feedback signal in electromyograph biofeedback: The relevance of attention” by Qualls and Sheehan. *J. Exp. Psychol. Gen.* 110 217–226. 10.1037/0096-3445.110.2.217 6454759

[B44] Travis F. Parim N. Shrivastava A. (2017). Higher theta and alpha1 coherence when listening to Vedic recitation compared to coherence during Transcendental Meditation practice. *Conscious. Cogn.* 49 157–162. 10.1016/j.concog.2017.02.002 28214765

[B45] Underhill E. (1955). *Mysticism: A Study in the Nature and Development of Man’s Spiritual Consciousness.* New York, NY: New American Library.

[B46] Ustinova Y. (2017). *Divine Mania: Alteration of Consciousness in Ancient Greece.* London: Routledge. 10.1177/0957154X20909306 32114829

[B47] Watts A. (1962). *The Joyous Cosmology: Adventures in the Chemistry of Consciousness.* New York, NY: Vintage Books.

[B48] Wittmann M. Neumaier L. Evrard R. Weibel R. Schmied-Knittel I. (2017). Subjective time distortion during near-death experiences: an analysis of reports. *Z. Anomal.* 17 309–320.

